# Effects of DASH diet with or without time-restricted eating in the management of stage 1 primary hypertension: a randomized controlled trial

**DOI:** 10.1186/s12937-024-00967-9

**Published:** 2024-06-17

**Authors:** Xiaoxin Zhou, Xiaoqian Lin, Jing Yu, Yi Yang, Hira Muzammel, Said Amissi, Valérie B. Schini-Kerth, Xun Lei, Pedro A. Jose, Jian Yang, Dan Shi

**Affiliations:** 1 Research Center for Metabolic and Cardiovascular Diseases, The Third Affiliated Hospital of Chongqing Medical University, Chongqing, China; 2 Department of Clinical Nutrition, The Third Affiliated Hospital of Chongqing Medical University, Chongqing, China; 3 Translational CardioVascular Medicine, Faculty of Pharmacy, UR 3074, University of Strasbourg, Strasbourg, France; 4 Department of Nutrition and Food Hygiene, School of Public Health, Chongqing Medical University, Chongqing, China; 5 Research Centre for Environment and Human Health, School of Public Health, Chongqing Medical University, Chongqing, China; 6 Department of Logistics Management Division, Chongqing Medical University, Chongqing, China; 7 Research Center for Medicine and Social Development, Collaborative Innovation Center of Social Risks Governance in Health, School of Public Health, Chongqing Medical University, Chongqing, China; 8 Department of Medicine and Department of Physiology and Pharmacology, Division of Renal Diseases and Hypertension, The George Washington University School of Medicine and Health Sciences, Washington, DC USA

**Keywords:** Time-restricted eating, Primary hypertension, Urinary Na^+^ excretion, Blood pressure

## Abstract

**Background:**

Time-restricted eating (TRE), a popular form of intermittent fasting, has shown benefits for improving metabolic diseases and cardiometabolic health. However, the effect of TRE in the regulation of blood pressure in primary hypertension remains unclear.

**Methods:**

A 6-week randomized controlled trial was conducted, in which a total of 74 stage 1 primary hypertensive patients without high-risk were randomly assigned to Dietary Approaches to Stop Hypertension (DASH) group (*n* = 37) or DASH + TRE group (*n* = 37). Participants in the DASH + TRE group were instructed to consume their food within an 8-h window. Scientific research platform in We Chat application was used to track participants. The primary outcome was blood pressure. The secondary outcomes included body composition, cardiometabolic risk factors, inflammation-related parameters, urinary Na^+^ excretion, other clinical variables and safety outcomes.

**Results:**

The reduction of systolic blood pressure and diastolic blood pressure were 5.595 ± 4.072 and 5.351 ± 5.643 mm Hg in the DASH group and 8.459 ± 4.260 and 9.459 ± 4.375 mm Hg in the DASH + TRE group. DASH + TRE group improved blood pressure diurnal rhythm. Subjects in DASH + TRE group had decreased extracellular water and increased urinary Na^+^ excretion. Furthermore, the decrease in blood pressure was associated with a reduction of extracellular water or increase in urinary Na^+^ excretion. In addition, safety outcomes such as nighttime hunger were also reported.

**Conclusion:**

Our study demonstrated that 8-h TRE + DASH diet caused a greater decrease in blood pressure in stage 1 primary hypertensive patients than DASH diet. This study may provide novel insights into the benefits of lifestyle modification in the treatment of primary hypertension.

**Trial registration:**

https://www.chictr.org.cn/ (ChiCTR2300069393, registered on March 15, 2023).

**Supplementary Information:**

The online version contains supplementary material available at 10.1186/s12937-024-00967-9.

## Introduction

Hypertension, a primary contributor to cardiovascular disease, overall mortality rates, and escalating healthcare expenditures, represents a significant and pressing global public health concern [1]. Based on epidemiological and clinical evidence, the 2017 ACC/AHA Guideline for the Prevention, Detection, Evaluation, and Management of High Blood Pressure in Adults updated the categorization of hypertension, defining stage 1 hypertension as a SBP of 130–139 mm Hg or a DBP of 80–89 mm Hg [2]. Subsequently, similar categorization was also recommended in other countries, including China [3]. Thess new guidelines may lead to a better control of blood pressure and more effective prevention of cardiovascular diseases.

The guidelines for blood pressure management clearly recommend patients with primary hypertension to choose lifestyle modification throughout the whole treatment process [2, 3]. Comprehensive lifestyle modifications, including healthy diet, weight loss, reduced sodium intake, increased physical activity, regular rest and limited alcohol intake, are proved to reduce blood pressure [2, 4–6]. The 2017 ACC/AHA Guideline recommends lifestyle modification alone (without pharmacotherapy) in patients with stage 1 hypertension with no atherosclerotic cardiovascular disease or estimated 10-year cardiovascular disease risk < 10% [2]. An estimated 22% of US adults meet the criteria for lifestyle modification alone, and many reported attempting behavior change [7]. Individuals with prehypertension and stage 1 hypertension can sustain multiple lifestyle modifications that improve the control of their blood pressure and reduce their cardiovascular disease risk [5, 8]. Lifestyle modification such as the combination of the Dietary Approaches to Stop Hypertension (DASH) diet and exercise even effectively lowers blood pressure in patients with resistant hypertension [9]. Thus, lifestyle modification has been shown to be important in the treatment of hypertension.

A healthy diet is an important component of lifestyle modification. Studies have reported that reasonable dietary modifications such as the DASH diet lower blood pressure and are recommended in the treatment of hypertensive subjects [10, 11]. Moreover, time-restricted eating (TRE), a unique form of intermittent fasting, has become very popular in recent years [12]. TRE does not require individuals to monitor their dietary intake or count calories during the eating “window” and has attracted increased attention for its beneficial effects in the management of some metabolic diseases such as obesity, type 2 diabetes, and nonalcoholic fatty liver disease [13–16]. Hypertension is also a metabolic disease. To our knowledge, there are only a few studies reporting the effect of TRE on blood pressure. TRE intervention improves cardiometabolic health, including significant decreases in systolic and diastolic blood pressures, in patients with metabolic syndrome receiving standard medical care [17]. TRE early in the day (8-h eating window from 7:00 to 15:00) also decreases diastolic blood pressure in adults with obesity [18]. However, the targets have been patients with metabolic diseases. To our knowledge, there is still no study reporting the effect of TRE in the treatment of primary hypertension.

We hypothesized that TRE would improve the blood pressure in subjects with primary hypertension. In the current study, we conducted a randomized control trial to investigate the effectiveness and safety of TRE in improving blood pressure in subjects with primary hypertension. It should be noted that only subjects with stage 1 hypertension were chosen in our study because of two reasons: 1) lifestyle modification alone are recommended for subjects with stage 1 hypertension without risks as aforementioned; and 2) TRE may prevent or delay the use of medications to treat the hypertension.

The aim of this 6 weeks study is to investigate whether there is a difference in blood pressure regulation in patients with stage 1 primary hypertension when implementing the DASH diet alone or in combination with TRE. Out of the 74 participants, 37 were randomly assigned to the DASH group and 37 were randomly assigned to the DASH + TRE group. Participants in the DASH + TRE group were instructed to consume their food within an 8-h window. Blood pressure, body composition, cardiometabolic risk factors, inflammation-related indicators, other clinical variables, blood pressure rhythm, urinary Na^+^ excretion, and safety outcomes were investigated.

## Methods

### Trial design and oversight

The primary objective for this study was to assess the role of TRE in stage 1 primary hypertension without high-risk. We conducted a randomized, controlled trial for 6 weeks, in which eligible trial participants were randomized to either a DASH group or a DASH combined with TRE group by random numbers table (please refer to the Randomization Procedure for a detailed description). This clinical trial was conducted at the Third Affiliated Hospital of Chongqing Medical University (Chongqing, China), approved and overseen by the Ethics Committees of the hospital, and conformed with the principles in the Declaration of Helsinki. Prior to enrolling participants, the trial was registered at the Chinese Clinical Trial Registry (ChiCTR2300069393). In addition, the entire trial process was managed on a scientific research platform, and all the data were inputted and checked by different researchers.

### Randomization procedure

Participants were randomized using a random numbers table to ensure unbiased allocation. The table was used to randomly determine row and column numbers, and then sequentially read two numbers from left to right and top to bottom. Numbers ranging from 01 to 74 were selected, and repeated selections of previously chosen numbers were avoided. The single number has been included in the dash group, while the double number has been included in the DASH + TRE group.

### Study participants

Trial participants were openly recruited from the Chongqing area between March 3 and April 17 2023 (time for recruiting) through online advertisements and distribution of posters. A total of 74 participants were recruited: 37 participants were randomly assigned to the DASH group, while 37 participants were randomly assigned to DASH + TRE group. The inclusion criteria were as follows: 1) 18–70 years old; 2) the first-time diagnosed stage 1 primary hypertension without high-risk (defining stage 1 hypertension as a SBP of 130–139 or a DBP of 80–89 mm Hg [2], without clinical complications, target organ damage or less than 3 cardiovascular risk factors); 3) healthy mental without communication barriers; 4) habitual eating time window ≥ 10 h; 5) regular sleeping schedule. The following participants were excluded from the study:1) history of diabetes, hypoglycemia, and secondary hypertension; 2) current smoking, serious liver dysfunction or chronic kidney disease, serious cardiovascular or cerebrovascular disease, blood or rheumatic immune diseases; severe respiratory or gastrointestinal diseases, orthopedic or endocrine diseases; uncontrolled neurological or psychiatric disorder; 3) cancer or infectious diseases, surgery in the 12 months before randomization; 4) pregnant, breast-feeding, or planned pregnancy; 5) used medications or supplements that could influence study outcomes. All patients provided written informed consent prior to enrollment.

### Sample size estimation

Since there are no previous clinical studies on the effect of TRE in primary hypertension, the results of the first four patients of each group decided by random numbers table in this experiment were used as preliminary experimental results for sample size estimation. The results showed that the mean ± standard deviation (SD) of SBP were 132.25 ± 6.08 (DASH group) and 123.00 ± 7.79 mmHg (DASH + TRE group), and the mean ± SD of DBP were 84.25 ± 3.10 (DASH group) and 75.00 ± 7.79 mmHg (DASH + TRE group). The power was set as 0.9 and α as 0.025, calculated by PASS software 15.0. Taking into account a 20% loss of subjects, at least 20 patients had to be recruited in each group.

### Intervention plan

During the 6-weeks trial, participants in the DASH + TRE group were instructed to consume their diet within an 8-h window during the daytime (9:00 a.m. to 5:00 p.m.) and fast the rest of the day [19]. Only water and energy-free drinks (e.g., decaffeinated coffee, and diet sodas) were allowed outside the eating window [20]. Participants in the control group were instructed to consume DASH diet, with food consumed over more than 8 h per day [21]. And no energy restrictions were imposed on two groups. In accordance with the recommendations from The Chinese Dietary Guidelines and personal workload, a suggested food and menu list at the beginning of the trial were provided [22, 23]. The daily recommended drinking water is at least 1350 ml. All participants were instructed to maintain a steady daily physical activity, and recorded exercise time and exercise type. Clinical features of baseline and exercise were shown in Supplemental Table 1 and Supplemental Table 2.

**Table 1:** Diurnal change of blood pressure determined by ambulatory blood pressure monitoring in the DASH + TRE group

	**Decrease of SBP (mm Hg)**	**Decrease of DBP (mm Hg)**	***P***
7 o'clock	7.714 ± 6.667	8.029 ± 5.993	< 0.001
10 o'clock	7.429 ± 4.642	5.400 ± 3.238	< 0.001
13 o'clock	6.057 ± 7.384	6.657 ± 9.480	< 0.001
16 o'clock	6.800 ± 5.503	6.400 ± 3.782	< 0.001
19 o'clock	6.943 ± 6.677	8.257 ± 7.180	< 0.001
22 o'clock	10.429 ± 9.121	7.857 ± 6.971	< 0.001

Data are shown as mean ± standard deviation (SD)

*SBP* Systolic blood pressure, *DBP* Diastolic blood pressure

**Table 2:** Changes in body composition, cardiovascular risk factors, inflammation related indicators and other clinical features in the DASH group

	**Variables**	**Pre-DASH**	**Post-DASH**	***P***
Body composition	Weight (kg)	61.000 (11.750)	60.000 (19.000)	> 0.05
	BMI (kg/m^2^)	24.896 ± 3.302	24.950 ± 4.132	> 0.05
	Total body water (L)	31.750 ± 2.590	31.578 ± 2.312	> 0.05
	Protein (Kg)	7.800 (1.375)	7.900 (1.300)	> 0.05
	Fat mass (Kg)	13.800 (3.000)	14.900 (3.300)	> 0.05
	Percentage of fat	0.227 ± 0.045	0.241 ± 0.049	> 0.05
	Skeletal muscle mass (Kg)	22.294 ± 3.917	22.552 ± 4.124	> 0.05
	Basal metabolic rate (Kcal)	1208.000 (169.000)	1220.000 (173.000)	> 0.05
	Visceral fat area (cm^2^)	93.106 ± 33.744	92.648 ± 30.877	> 0.05
	Bone mineral content (Kg)	2.495 (0.618)	2.500 (0.830)	> 0.05
	Waist hip ratio	0.823 ± 0.054	0.829 ± 0.051	> 0.05
Cardio-vascular risk factors	AopA1/ApoB	1.620 (0.485)	1.670 (0.550)	> 0.05
	ApoB (g/L)	0.969 ± 0.261	0.898 ± 0.223	> 0.05
	AopA1 (g/L)	1.535 ± 0.161	1.521 ± 0.172	> 0.05
	TG (mmol/L)	2.280 (1.560)	1.850 (1.520)	> 0.05
	TC (mmol/L)	4.540 (1.295)	4.650 (1.010)	> 0.05
	HDL-C (mmol/L)	1.337 ± 0.245	1.380 ± 0.267	> 0.05
	LDL-C (mmol/L)	2.750 (0.800)	2.750 (0.820)	> 0.05
	Fasting blood glucose (mmol/L)	5.075 ± 0.108	5.153 ± 0.128	> 0.05
	Insulin (μU/mL)	12.530 (14.280)	8.750 (13.800)	> 0.05
	HOMA-IR	3.023 ± 3.231	2.261 ± 2.439	> 0.05
Inflammation-related parameters	White blood cell count (10^9^)	6.650 (1.775)	5.900 (1.200)	> 0.05
	Neutrophil percentage% (%)	59.934 ± 7.183	59.241 ± 5.684	> 0.05
	C reactive protein (mg/L)	2.000 (0.000)	2.030 (0.400)	> 0.05
	Interleukin 6 (pg/mL)	2.560 (1.695)	3.030 (20.215)	> 0.05
Other clinical variables	ALT (U/L)	18.300 (11.200)	15.800 (5.100)	> 0.05
	AST/ALT	1.150 (0.485)	1.180 (0.430)	> 0.05
	C-glutamyl transpeptidase (U/L)	21.600 (14.200)	25.000 (26.100)	> 0.05
	AST(U/L)	20.900 (5.850)	19.400 (6.800)	> 0.05
	Serum total protein(g/L)	75.400 (5.100)	73.500 (4.400)	> 0.05
	Alkaline Phosphatase (U/L)	82.300 (28.450)	77.400 (32.600)	> 0.05
	Lactate dehydrogenase (U/L)	191.800 (27.300)	183.950 (31.500)	> 0.05
	Globulin (g/L)	29.200 (4.100)	28.300 (5.900)	> 0.05
	Uric acid (μmoI/L)	300.446 ± 88.022	292.626 ± 83.851	> 0.05
	Urea nitrogen (mmol/L)	6.676 ± 1.350	6.022 ± 1.206	= 0.05
	Creatinine (μmoI/L)	58.900 (16.200)	64.400 (17.800)	> 0.05
	Red blood cell count (10^12^)	4.590 ± 0.360	4.423 ± 0.416	> 0.05
	Platelets count (10^9^)	235.813 ± 64.486	236.091 ± 62.841	> 0.05
	Lymphocyte percentage (%)	31.888 ± 5.968	31.032 ± 5.188	> 0.05

Normal distribution data are expressed as mean ± SD, and non-normal data are expressed as median (interquartile range)

*n* = 37:27 for most variables between two groups, n of IL-6 and blood routine for Post-DASH is 18 and 22 respectively, n of a few variables are between 20–26, a few outliers were excluded

*BMI* Body mass index, *TG* Total glyceride, *TC* Total cholesterol, *HDL-C* High-density lipoprotein cholesterol, *LDL-C* Low-density lipoprotein cholesterol, *HOMA-IR* Homeostatic model assessment of insulin resistance, *ALT* Alanine aminotransferase, *AST* Aspartate transaminase

### Assessments and adherence

Dietary counseling was provided by trained nutritionists and physicians. The participants could contact nutritionists or physicians at any time and any place though online communication and received dietary guides and questionnaires Participants have two options based on their personal habits of using the We Chat application. The first option is to utilize the scientific research platform, a mini-program embedded within the We Chat application, while the other is to directly engage in group conversations with nutritionists and physicians via We Chat. Therefore, there is no substantial difference between the two methods. Through above two methods, participants shared images of their meals, which were then assessed and evaluated by trained nutritionists. Four participants opted for the mini-program, whereas 70 patients chose the group conversation function. One investigator checked and assessed the daily dietary record of the participants based on the nutritional content shown in Chinese Food Composition Tables, which was double-checked by another investigator [24]. Another nutritionist assessed, calculated and inputted the quantities and energy supply of the three major nutrients (carbohydrates, protein and fat) based on the types and quantities of foods consumed by the participants as shown in Supplemental Table 3. Participants who have not complied with the DASH rule or eating time more than 8 h for DASH + TRE group were considered “not adhered”. Adherence to the dietary program was defined by the number of days participants met the dietary requirements, and the adherence ratio was shown in results section and Supplemental Table 4.

**Table 3:** Effects of DASH and TRE Diets on body composition, cardiovascular risk factors, inflammation-related variables and other clinical features

	**Variables**	**Pre-(DASH + TRE)**	**Post-(DASH + TRE)**	***P***
Body composition	Weight (kg)	59.980 (9.730)	55.930 (10.94)	0.029
	BMI (kg/m^2^)	23.610 (2.790)	22.310 (2.560)	0.005
	Total body water (L)	31.162 ± 1.716	30.251 ± 1.700	0.025
	Protein (Kg)	7.900 (1.200)	8.300 (1.200)	> 0.05
	Fat mass (Kg)	12.484 ± 2.233	11.224 ± 2.293	0.019
	Percentage of fat	0.207 ± 0.042	0.192 ± 0.042	> 0.05
	Skeletal muscle mass (Kg)	22.778 ± 3.686	22.403 ± 3.458	> 0.05
	Basal metabolic rate (Kcal)	1241.000 (197.500)	1226.000 (197.500)	> 0.05
	Visceral fat area (cm^2^)	97.930 ± 27.513	100.678 ± 27.435	> 0.05
	Bone mineral content (Kg)	2.920 ± 0.698	2.910 ± 0.645	> 0.05
	Waist hip ratio	0.810 (0.100)	0.810 (0.080)	> 0.05
Cardio-vascular risk factors	AopA1/ApoB	1.855 (0.663)	1.615 (0.660)	> 0.05
	ApoB (g/L)	0.840 (0.328)	0.910 (0.310)	> 0.05
	AopA1 (g/L)	1.464 ± 0.219	1.545 ± 0.134	> 0.05
	TG (mmol/L)	1.025 (0.608)	1.300 (0.645)	> 0.05
	TC (mmol/L)	4.552 ± 1.005	4.906 ± 0.641	> 0.05
	HDL-C (mmol/L)	1.452 ± 0.338	1.445 ± 0.229	> 0.05
	LDL-C (mmol/L)	2.711 ± 0.909	2.956 ± 0.540	> 0.05
	Fasting blood glucose (mmol/L)	5.084 ± 0.636	4.957 ± 0.652	> 0.05
	Insulin (μU/mL)	6.290 (4.820)	7.530 (2.705)	> 0.05
	HOMA-IR	1.701 ± 1.396	1.899 ± 1.073	> 0.05
Inflammation-related variables	White blood cell count (10^9^)	7.706 ± 1.615	6.250 ± 0.636	> 0.05
	Neutrophil percentage% (%)	68.140 ± 7.205	64.200 ± 0.707	> 0.05
	C reactive protein (mg/L)	2.000 (0.000)	2.000 (0.000)	> 0.05
	Interleukin 6 (pg/mL)	2.000 (0.260)	2.540 (1.108)	> 0.05
Other clinical variables	Alanine aminotransferase (U/L)	15.800 (12.200)	18.800 (12.200)	> 0.05
	AST/ALT	1.370 (0.605)	1.040 (0.395)	> 0.05
	C-glutamyl transpeptidase (U/L)	18.300 (14.750)	26.900 (17.650)	> 0.05
	AST(U/L)	20.300 (7.450)	22.300 (6.650)	> 0.05
	Serum total protein(g/L)	75.400 ± 6.973	75.822 ± 4.258	> 0.05
	Alkaline Phosphatase (U/L)	84.000 (26.850)	85.000 (26.350)	> 0.05
	Lactate dehydrogenase (U/L)	182.150 (40.150)	182.600 (33.000)	> 0.05
	Globulin (g/L)	27.713 ± 3.652	28.341 ± 2.773	> 0.05
	Uric acid (μmoI/L)	350.076 ± 76.183	372.111 ± 80.169	> 0.05
	Urea nitrogen (mmol/L)	4.800 (1.750)	5.200 (1.200)	> 0.05
	Creatinine (μmoI/L)	63.900 (19.900)	72.750 (15.25)	> 0.05
	Red blood cell count (10^12^)	4.036 ± 0.835	5.335 ± 0.134	> 0.05
	Platelets count (10^9^)	233.200 ± 17.441	210.000 ± 25.456	> 0.05
	Lymphocyte percentage (%)	21.360 ± 9.582	28.100 ± 1.414	> 0.05

Normal distribution data are expressed as mean ± SD, and non-normal data are expressed as median (interquartile range)

*n* = 37:37 for most variables between two groups, n of few variables are between 30–36, n of  a few outliers were excluded

*BMI* Body mass index, *TG* Total glyceride, *TC* Total cholesterol, *HDL-C* High-density lipoprotein cholesterol, *LDL-C* Low-density lipoprotein cholesterol, *HOMA-IR* Homeostatic model assessment of insulin resistance, *ALT* Alanine aminotransferase, *AST* Aspartate transaminase

### Measurement of trial outcomes

The primary outcome was the change in blood pressure. The secondary outcomes were changes in body composition, cardiometabolic risk factors, inflammation-related indicators, other clinical variables, blood pressure rhythm, urinary Na^+^ excretion, and safety outcomes. Physical activity, Pittsburgh Sleep Quality Index (PSQI) and score of life events were also evaluated.

All variables, including cardiometabolic risk factors, inflammation-related indicators and other clinical variables (liver and renal functions, etc.) were measured at baseline and the end of 6-week follow-up visits. Body weight and body composition, including total body water, extracellular water and intracellular water, fat mass, skeletal muscle mass, and basal metabolic rate etc., were evaluated by the direct segmental multifrequency bioelectrical impedance analysis method (Inbody S10; Biospace, Seoul, Korea). Waist circumference and hip circumference were measured by the same investigator to the nearest 0.1 cm for the calculation of the waist-to-hip ratio (WHR).

Urinary Na^+^ was measured by Na^+^ colorimetry (Nanjing Jiancheng Bioengineering Institute, Nanjing, China). Total nocturnal sodium excretion was calculated as the urine sodium concentration × nocturnal urine volume [25]. The intra-assay coefficient of variability (CV) was 1.5% while the inter-assay CV was less than 5.0%. It should be noted that the participants in the DASH group were only required to collect their urine overnight, rather than over a full 24-h period, because we tried to increase the comfortable experience of control subjects. Nighttime urine was collected as the following: participants were instructed to ensure continuous and regular sleeping before urine collection for at least 7 days. Starting at 23:00 on the day before urine collection, participants were instructed to empty their bladders and then go to sleep. After sleeping continuously for 8 h, participants collected the first morning urine. For the 24-h urine collection: participants followed the same instructions for continuous and regular sleeping, and then urine was collected over a 24-h period.

Blood pressure was measured by the same trained researcher with the same automatic device (Yuyue Medical Equipment Co., Ltd., Danyang, China). Participants kept the right upper arm at the heart level in a seated position, after at least a 5 min rest in a quiet room [26]. The average of three measurements was used for analyses. Morning blood pressure was measured at 7:00 a.m., and ambulatory blood pressure monitoring was performed at 7:00, 10:00, 13:00, 16:00, 19:00, and 22:00. The differences between the blood pressure levels in the morning and at night were recorded to determine the blood pressure fluctuations [27].

In addition, the Pittsburgh Sleep Quality Index (PSQI) was used to evaluate the sleeping quality of the participants; a high PSQI score is indicative of worse sleep quality [28, 29]. The score of life events scale was used to calculate all the participants' life events before and after the intervention [30]. The type, frequency, and duration of exercise were also recorded, and the PSQI and life events were used to exclude the possible effect on the patient's blood pressure by sleeping or life events.

Safety outcomes (nighttime hunger, nocturnal hypoglycemia, stomachache, nausea, vomiting, diarrhea, constipation, palpitations, chest tightness or chest pain, upper respiratory tract infection, other infections, pain in other areas, fatigue) and withdrawal from study due to adverse reactions were assessed by daily communication.

Data of blood pressure, urinary Na^+^, weight, safety outcomes, PSQI, blood sample collection and life events were obtained within ± 3 days at the baseline (day 0) and final follow-up (day 42nd).

### Statistical analysis

All analyses were performed with SPSS version 20.0 (SPSS, Chicago, USA). The normal distributed data were expressed as mean ± standard error (SE) or mean ± standard deviation (SD); the non-normal distributed data were expressed as median (interquartile range). Normal distribution of data was tested by both Kurtosis and Skewness Test. The two-tailed *t* test was used to compare two independent normally distributed samples, while non-parametric tests were performed to compare non-normally distributed data. In addition, one-way ANOVA was used to compare more than two groups. Pearson correlation analysis was performed to evaluate the correlation between each variable. A value of two-side *P* < 0.05 was considered statistically significant.

## Results

### Trial participants

From March 3, 2023 to May 29, 2023 (time for the entire experiment), a total of 74 subjects were randomly assigned to either DASH group (37 participants) or DASH + TRE group (37 participants). The study diagram is shown in Supplemental Fig. 1. The mean ± SD age of the participants was 48.34 ± 7.46 years, and the mean ± SD body mass index (BMI) was 24.35 ± 2.80 kg/m^2^. The characteristic of the participants at baseline were similar between the two groups, including age, weight, BMI, SBP, DBP, body composition (such as total body water, fat mass, skeletal muscle mass, basal metabolic rate, waist-hip ratio), fasting lipid and blood glucose levels, liver and renal functions, and night urine Na^+^ excretion, etc. (Supplemental Table 1). Physical activity, PSQI, and score of life events were also similar between the two groups over the 6 weeks of the trial (Supplemental Table 2; Supplemental Figs. 2 and 3).

**Fig. 1:**
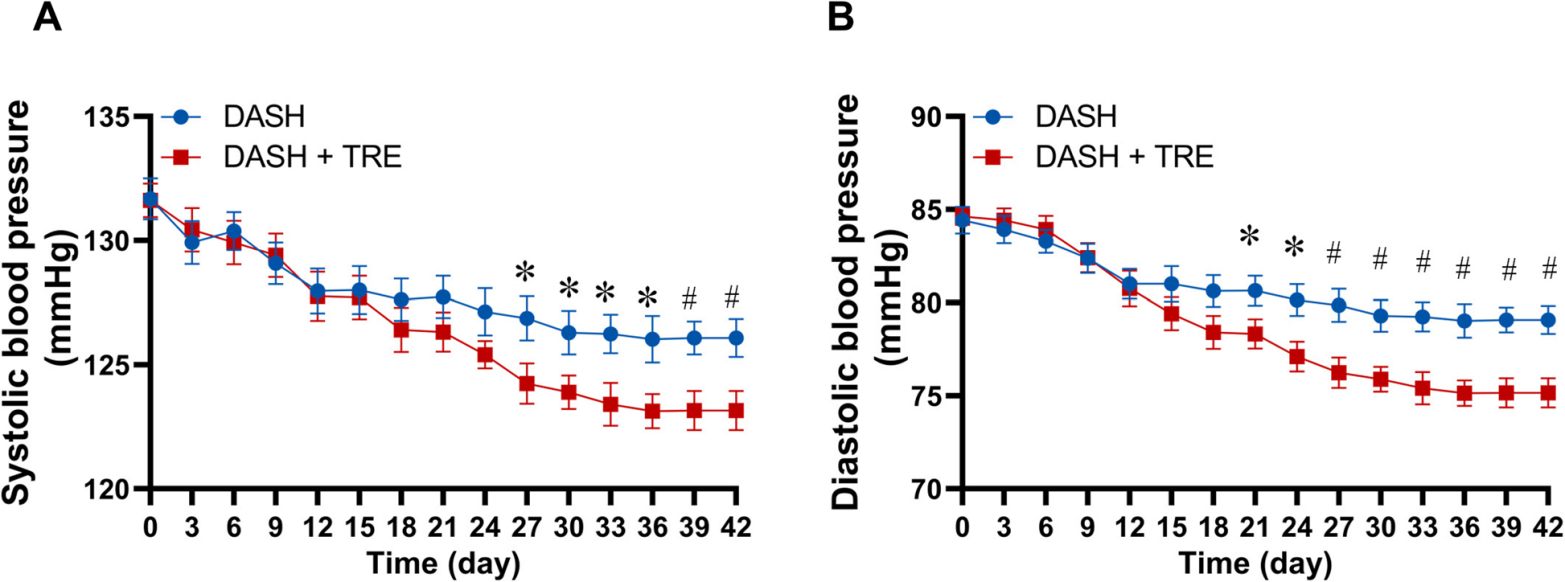
Dynamic graphs of morning systolic blood pressure (**A**) and diastolic blood pressure (**B**) in patients in DASH group and DASH + TRE group. *n* = 37:37, *p < 0.05, #*p* < 0.01 *vs* DASH group. DASH, dietary approaches to stop hypertension; TRE, time-restricted eating

**Fig. 2:**
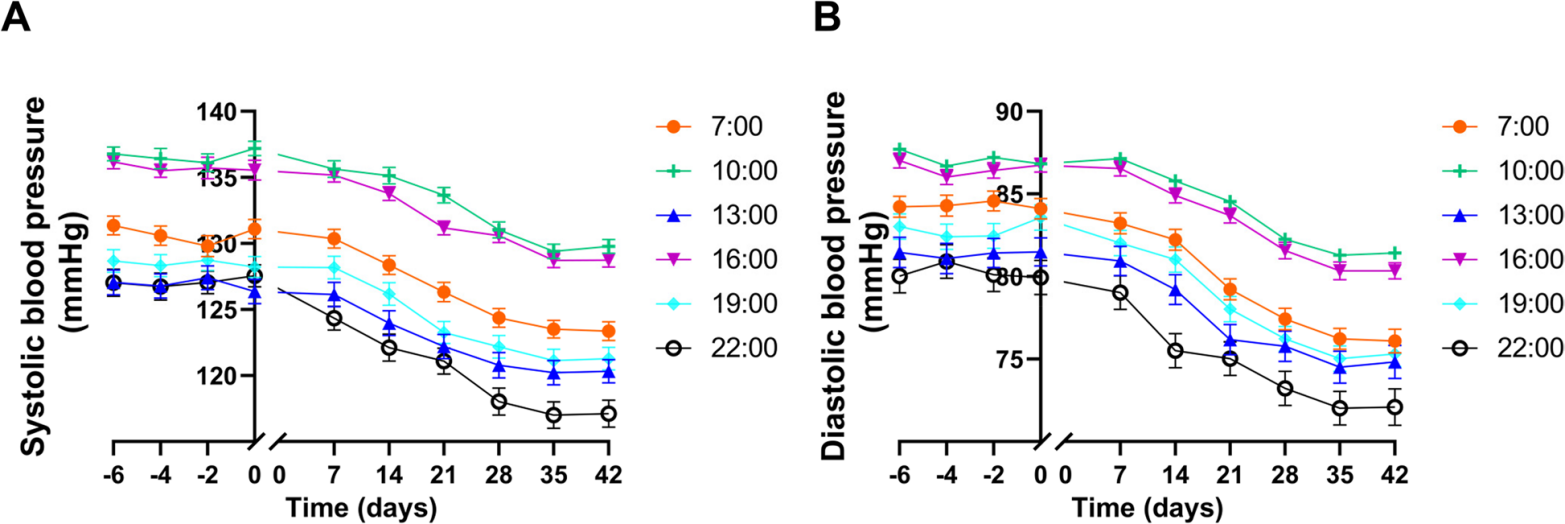
Blood pressure rhythm determined by ambulatory blood pressure monitoring in the DASH + TRE group. **A** Systolic blood pressure; **B** Diastolic blood pressure. *n* = 37 participants/point, *p < 0.05 *vs* 0 week, ^#^*p* < 0.01 *vs* 0 week. -2 represents 2 days before the start of the experiment, and the rest are similar. DASH, dietary approaches to stop hypertension; TRE, time-restricted eating

**Fig. 3:**
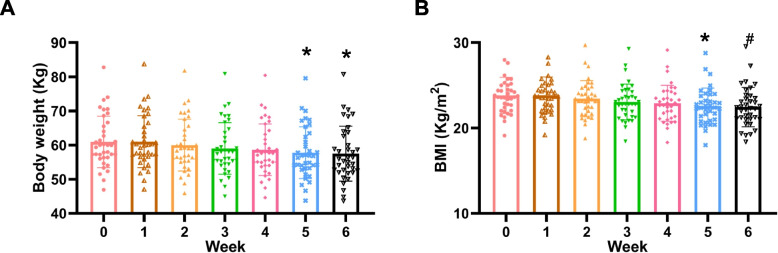
Body weight (**A**) and BMI (**B**) in the DASH + TRE group. *n* = 37/time point, *p < 0.05 *vs* 0 week, ^#^*p* < 0.01 *vs* 0 week. DASH, dietary approaches to stop hypertension; TRE, time-restricted eating

During the 6 weeks intervention, only the subjects who adhered to both the prescribed caloric intake and eating time period could participate in the final follow-up. Among 74 participants, 62 participants (83.78%) completed the entire 6-weeks diet record of three meals per day. Based on the photos of three meals per day uploaded to the scientific research platform or instant chat communication group as records, the 8-h time restriction was followed. The contents of major nutrients (carbohydrate, lipid and protein) and the energy percentage from the major nutrients were both similar between the two groups during the 6 weeks trial (Supplemental Table 3).

### Role of TRE on blood pressure

#### Morning blood pressure lowering in the two groups

The SBP and DBP at 7 o’clock a.m. in both groups gradually decreased with the intervention time. However, the blood pressure of the DASH + TRE group decreased more than that of the DASH group, which was evident at the 27th day for SBP or at the 21st day for DBP, and maintained up to the 42nd day (Fig. 1). The reduction of SBP and DBP from baseline to the 6th week were 5.595 ± 4.072 mmHg and 5.351 ± 5.643 mmHg in the DASH group and 8.459 ± 4.260 mmHg and 9.459 ± 4.375 mmHg in the DASH + TRE group. Besides the difference between two groups at 42nd day were 2.920 mmHg and 3.919 mmHg respectively (Fig. 1).

#### Improvement of the blood pressure diurnal rhythm in the DASH + TRE group

To further explore the effect of TRE on blood pressure, we examined blood pressure diurnal rhythm (7:00, 10:00, 13:00, 16:00, 19:00, and 22:00) of TRE participations using ambulatory blood pressure monitoring. The blood pressure was reduced at indicated time point in this group, as shown in Table 1 and Fig. 2. The decrease in SBP at 22 o'clock compared with 10 o'clock increased from 6.979% to 9.753% in the DASH + TRE group after intervention, while the decrease in DBP at 22 o'clock compared with 10 o'clock increased from 7.869% to 11.473% (Calculation method: for each time point, the decrease was obtained by subtracting the post-intervention blood pressure from the pre-intervention blood pressure, and then dividing the result by the pre-intervention blood pressure). These indicated that DASH + TRE intervention improved the blood pressure diurnal rhythm.

#### Comparison of BMI, body composition, cardiometabolic risk factors, inflammation-related parameters and other clinical variables before and after intervention in the two groups

**Fig. 4:**
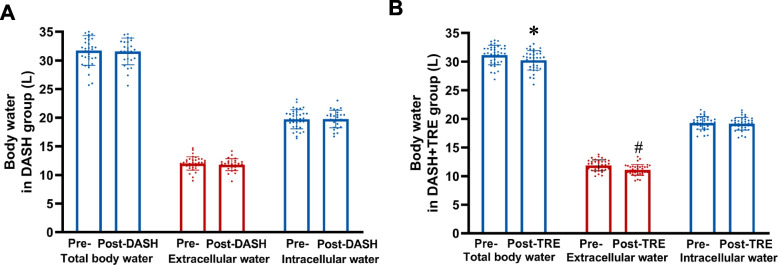
Body water before and after intervention in the two groups. **A** Total body water, extracellular water and intracellular water in the DASH group, *n* = 36:27, *p* > 0.05. **B** Total body water, extracellular water and intracellular water in the DASH + TRE group, *n* = 37:37, * *p* < 0.05, ^#^ *p* < 0.01

In the DASH + TRE group, both weight and BMI decreased, which became significantly evident at the 5th week of intervention (Fig. 3). On the contrary, there was no difference in weights before and after intervention in the DASH group (Table 2). 40 variables, including body composition, cardiometabolic risk factors, inflammation-related parameters and other clinical variables, were compared before and after intervention in the two groups. In the DASH group, all variables were unchanged before and after intervention (Table 2). However, several of variables such as weight, BMI, and body fat mass were significantly decreased in the DASH + TRE group (Table 3 and Fig. 4). Since total body water, consisting of extracellular water and intracellular water, is part of the regulation of blood pressure, total body water was also measured. Our results showed that both extracellular water and intracellular water were unchanged before and after DASH intervention alone (Fig. 4). However, total body water and extracellular body water were decreased before and after combined DASH and TRE intervention, while intracellular body water was unchanged (Table 3 and Fig. 4).

### Role of TRE on urinary Na^+^ excretion

#### Changes in urinary Na^+^ excretion in the two groups

**Fig. 5:**
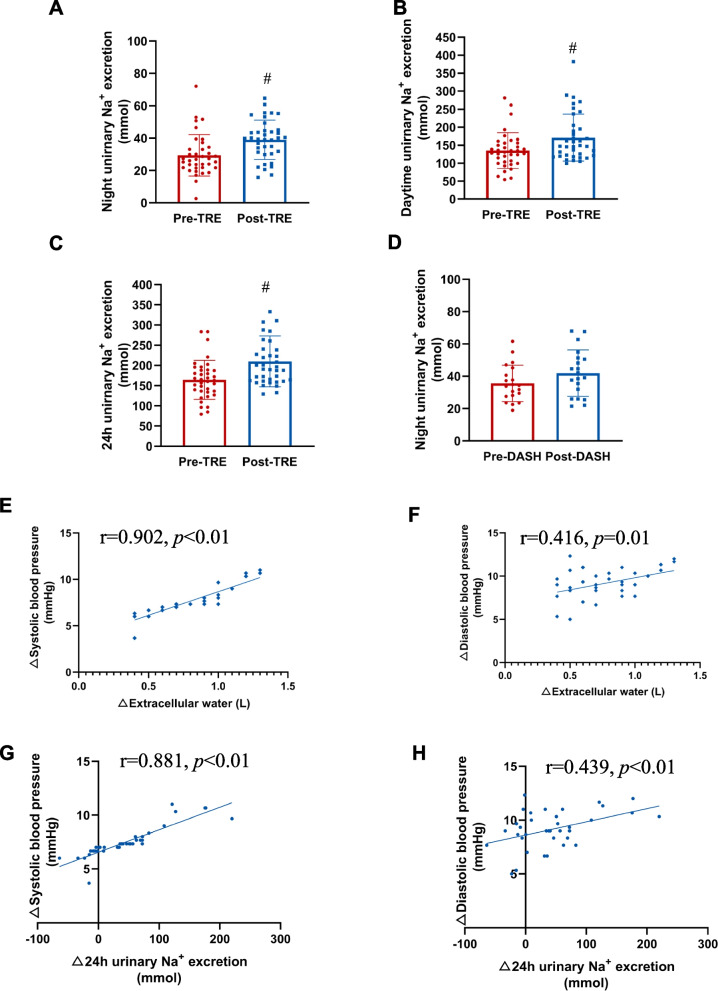
Correlation between the decrease in systolic and diastolic blood pressure, and the decrease in total body water and increase in urinary Na^+^ excretion in DASH + TRE group. **A** Night urinary Na^+^ excretion before and after intervention in DASH + TRE group, *n* = 37, ^#^*p* < 0.01. **B** Daytime urinary Na^+^ excretion before and after intervention in DASH + TRE group, *n* = 37, ^#^*p* = 0.01. **C** 24-h urinary Na^+^ excretion before and after intervention in DASH + TRE group, *n* = 37, ^#^*p* < 0.01. **D** Night urinary Na^+^ excretion before and after intervention in DASH group, *n* = 20, *p* > 0.05. **E** Correlation between the decreased SBP and extracellular water in DASH + TRE group, *n* = 37, *r* = 0.902, *p* < 0.01. **F** Correlation between decreased DBP and extracellular water in DASH + TRE group, *n* = 37, *r* = 0.416, *p* = 0.01. **G** Correlation between decreased SBP and increased 24 h urinary Na.^+^ excretion in DASH + TRE group, *n* = 37, *r* = 0.881, *p* < 0.01. **H** Correlation between decreased DBP and increased 24 h urinary Na + excretion in DASH + TRE group, *n* = 37, *r* = 0.439, *p* < 0.01

In order to clarify whether the antihypertensive effect of TRE was associated with sodium homeostasis, the urinary Na^+^ excretion was measured before and after intervention. We found the urinary Na^+^ excretion after DASH + TRE intervention had a significant increase in the whole day, including daytime and nighttime (Fig. 5A-C). However, after 6 weeks there was no change of night urinary Na^+^ excretion in the DASH group (Fig. 5D). The participants in the DASH group were only required to collect their urine overnight, rather than over a full 24-h period, because we tried to increase the comfortable experience of control subjects.

#### Correlation between decreased blood pressure, reduced total body water and increased urinary sodium excretion

We found that the decreases of SBP and DBP were associated with the decrease in the extracellular water in the DASH + TRE group (Fig. 5E and F). Moreover, the decreases of blood pressures also correlated with the increase in urinary Na^+^ excretion in this group (Fig. 5G and H). These results indicated that the increase in Na^+^ excretion may be the mechanism for the TRE-mediated decrease in blood pressure. It should be noted that we did not analyze the correlation between the reduction in blood pressure and changes of extracellular water and urinary Na^+^ in the DASH group, because there were no statistically significant changes in extracellular water and urinary Na^+^ in the DASH group before and after intervention (Figs. 4 and 5).

#### Safety and adherence outcomes

In the 6 weeks of the diets, no serious adverse events were observed; however, 8 of 74 subjects reported at least one mild adverse event, with nighttime hunger most frequently reported. In the DASH group, nighttime hunger, upper respiratory tract infection, and pain in other areas of the body occurred (Supplemental Table 5), while nighttime hunger, nausea, and diarrhea occurred in the DASH + TRE group (Supplemental Table 6). Nighttime hunger was more frequent in the DASH + TRE group (5.405%) than the DASH group (2.600%). Two participants in the DASH + TRE group and one participant in the DASH group experienced hunger while sleeping in the early stages of the trial without hypoglycemia. In addition, the degree of hunger was mild, and the participants got better after taking energy-free drink or falling asleep quickly. The remaining symptoms were not relevant to this study. Besides the adherence ratio were 90.938% for DASH group while 90.476% for DASH + TRE group, which were shown in Supplemental Table 4.

## Discussion

Regular meal timing influences the body’s internal circadian clock and enables physiological processes to be performed at the optimal time [31]. Recently, TRE, which belongs to intermittent fasting schedule, has become an effective and popular method in the regulation of body weight and metabolic diseases such as diabetes [14]. Moreover, epidemiological and animal studies have also shown that TRE can improve the cardiometabolic profile and cardiovascular diseases, such as atherosclerosis [32]. However, the effect of TRE in the regulation of blood pressure in primary hypertension is still unclear. The current reports on the effect of TRE on blood pressure were only in patients with metabolic syndrome and obesity [17, 18]. In our randomized controlled trial, both DASH and 8-h TRE + DASH regimens caused a decrease in blood pressure. However, the 8-h TRE + DASH regimen produced a greater decrease in blood pressure than the DASH regimen. Moreover, we also found TRE had a greater contribution to reduce blood pressure at 22:00 o’clock.

The pathogenesis of hypertension is complicated, which includes arterial function, abnormal body sodium homeostasis, insulin resistance, and inflammation. In this study, we explored the possible reasons for the TRE-mediated regulation of blood pressure. After comparing 40 variables, including body composition, cardio-vascular risk factors, inflammation-related and other clinical variables before and after intervention, we found that extracellular body water decreased in the DASH + TRE group. Previous studies have shown the body water, including total body water, intracellular water and extracellular water are positively correlated with blood pressure in different patients, including those with chronic kidney disease [33], those on hemodialysis and those with primary hypertension [34, 35]. Blood pressure is sensitive to extracellular body water; extracellular water overhydration was significantly greater in patients whose blood pressures increased post dialysis [36, 37]. We also found that the association between decreased blood pressure and reduced extracellular body water in DASH + TRE group. Thus, extracellular water may be involved in the regulation of blood pressure by TRE. Moreover, the reduction in systolic and diastolic blood pressure of participants in the DASH + TRE group significantly exceeded that of the DASH group on day 27 and day 21 (as shown in Fig. 1), while weight loss in the DASH + TRE group occurred in the fifth week (as shown in Fig. 3). This indicates that the decrease in blood pressure among participants in the DASH + TRE group preceded the reduction in body weight. Therefore, we speculate that the blood pressure-lowering effect of TRE is not attributed to weight loss. These findings warrant further validation in subsequent prospective studies.

Sodium homeostasis plays a vital role in the regulation of blood pressure; Na^+^ accumulation inevitably leads to water retention [38–40]. We measured urine Na^+^ excretion of the participants in our study. Our results showed that the DASH + TRE group had a marked increase in night urinary Na^+^ excretion relative to the DASH group. Moreover, the 24 h urinary Na^+^ excretion after 6 weeks was increased in the DASH + TRE group, which is considered to be in accord with the TRE-mediated important effect, the normalization of the circadian clock. Studies have shown that time-restricted feeding exerts its physiological functions by regulating the circadian clock [41, 42]. We also found that the correlation between decrease in blood pressure and increased urinary sodium excretion in DASH + TRE group. In addition, previous animal experiments have shown that time-restricted feeding decreases serum creatinine, increases creatinine clearance, and reduces blood pressure in two mouse models of hypertension [43]. These studies indicated that Na^+^ excretions are important mechanisms of TRE-mediated regulation of blood pressure.

We paid considerable attention to the adverse effects and safety of TRE. In this trial, only few had nighttime hunger. Two patients experienced hunger during sleeping time in the early stages of the trial, suggesting that patients can tolerate TRE by establishing regular eating habits. Since there was no nighttime hunger before starting TRE, this could be considered to be related to TRE. However, no hypoglycemia occurred, implying that TRE is relatively safe, and hunger may be based on lack of satiety. The primary novelty of this study lies in its discovery of the effective blood pressure-lowering effects of TRE in patients with stage 1 primary hypertension. Our results demonstrate that TRE holds strong translational potential as a lifestyle intervention for stage 1 hypertensive patients. Additionally, this study concurrently identified that alterations in water and Na^+^ excretion may be one of the key mechanisms through which TRE regulates blood pressure.

The strengths of this study include two intervention methods of DASH and DASH + TRE, while recording other major living factors such as sports, PSQI for sleeping assessment and score of life events, and random control method was used to conduct research. We specifically chose stage 1 patients with low risk according, since guidelines recommend lifestyle interventions for such individuals. Each participant had been guided by at least one doctor and one nutritionist, and timely chat group have been set up at any time to interact with them at any time. Moreover, the nutritionist used pictures sent by the participants to assess in order to make sure on authenticity.

There are also some limitations in this study. First, the 6-week time of follow-up does not allow us to observe a longer effect of TRE in the regulation of blood pressure. Second, the rhythmicity of the variables was not measured. Therefore, we could not be completely sure whether the beneficial effect of TRE on blood pressure was related to the circadian clock. Third, in order to ensure patients’ rest during sleeping time without affecting their normal biological clock rhythm, we did not measure blood pressure between 22:00 at night to 7:00 in the morning. We look forward to subsequent studies using 24-h ambulatory blood pressure monitory. After the obvious antihypertensive effect of the DASH + TRE group has been observed (Fig. 1), we hope to explore that for which time period is more obviously for the TRE to reduce blood pressure, so we only focused on the blood pressure rhythm of the DASH + TRE group. Moreover, the trial is subject to inherent biases that cannot be avoided, and blinding was not feasible in this study due to the nature of the interventions. In addition, there may be occasional instances of potential deception in self-reported dietary intake by individual participants. Above limitations deserve to be improved in subsequent research.

## Conclusions

In summary, our current trial found that 8-h TRE + DASH regimen produced a greater blood pressure lowering in stage 1 primary hypertensive patients than the DASH regimen. These studies may advance our understanding of the role of TRE in the control of primary hypertension and provide novel insights into the field of translational medicine, especially in the design of new lifestyle modifications in the treatment of hypertension.

Our findings suggest that TRE, a unique form of intermittent fasting, exerts a beneficial effect in the regulation of blood pressure in subjects with primary hypertension. The increase in urinary Na^+^ excretion may have mediated the beneficial effect of TRE on blood pressure. These results suggest that as a promising lifestyle modification, it may be worthwhile to apply TRE regimen in the control of primary hypertension. However, more randomized controlled trials with more participants are needed to confirm the beneficial effect of TRE in primary hypertension. It is also important to determine, in the future, the underlying mechanisms of the beneficial effect of TRE in primary hypertension.

### Supplementary Information

Supplementary Material 1: Supplemental Table 1. Comparison of clinical features between DASH and DASH+TRE groups at baseline. Supplemental Table 2. Times of aerobic and resistance exercise per week in DASH and DASH+TRE groups. Supplemental Table 3. Main nutrient composition between DASH and DASH+TRE groups after 6 weeks intervention. Supplemental Table 4. Adherence to the dietary program. Supplemental Table 5. Adverse events in the DASH group. Supplemental Table 6. Adverse events in the DASH+TRE group.

Supplementary Material 2: Supplemental Figure 1. Participant flow diagram. Participants were randomized to either DASH (*n*=37) or DASH+TRE (*n*=37).Supplemental Figure 2. Pittsburgh sleep quality index (PSQI) in the two groups. A. PSQI before and after intervention in DASH group, *n*=26:26, *p* >0.05; B. PSQI before and after intervention in DASH+TRE group, *n*=37:37, *p* >0.05. Supplemental Figure 3. Life Events Score in the two groups. A. Life Events Score before and after intervention in DASH group, *n*=26:26, *p* >0.05; B. Life Events Score before and after intervention in DASH+TRE group, *n*=37:37, *p* >0.05. Supplemental Figure 4. Body weight (A) and BMI (B) in the DASH group. *n*=36/time point, DASH, dietary approaches to stop hypertension.

## Data Availability

The protocols of this article will be made available upon reasonable request from the first or corresponding authors.

## Abbreviations

BMI: Body mass index
CV: Coefficient of variation
DASH: Dietary Approaches to Stop Hypertension
DBP: Diastolic blood pressure
PSQI: Pittsburgh sleep quality index
SBP: Systolic blood pressure
TRE: Time-restricted eating
WHR: Waist-to-hip ratio
