# Supplementary material for: Effects of DASH diet with or without time-restricted eating in the management of stage 1 primary hypertension: a randomized controlled trial
Source: Nutr J. 2024 Jun 17;23:65. doi: 10.1186/s12937-024-00967-9 (PMC11181626; doi:10.1186/s12937-024-00967-9)
Supplement: Supplementary file 1 — Supplementary Material 1: Supplemental Table 1. Comparison of clinical features between DASH and DASH+TRE groups at baseline. Supplemental Table 2. Times of aerobic and resistance exercise per week in DASH and DASH+TRE groups. Supplemental Table 3. Main nutrient composition between DASH and DASH+TRE groups after 6 weeks intervention. Supplemental Table 4. Adherence to the dietary program. Supplemental Table 5. Adverse events in the DASH group. Supplemental Table 6. Adverse events in the DASH+TRE group. [file 12937_2024_967_MOESM1_ESM.doc]

**Supplemental Table 1. Comparison of clinical features between DASH and DASH+TRE groups at baseline**

| **Variables** | **DASH** | **DASH+TRE** | ***P*** |
| --- | --- | --- | --- |
| Age (years) | 49.194±7.123 | 47.514±7.784 | >0.05 |
| Weight (kg) | 61.000 (11.750) | 59.980 (9.730) | >0.05 |
| BMI (kg/m^2^) | 24.896±3.302 | 23.815±2.119 | >0.05 |
| SBP (mmHg) | 131.676±5.017 | 131.622± 4.132 | >0.05 |
| DBP (mmHg) | 85.000 (8.000) | 85.000 (3.500) | >0.05 |
| Total body water (L) | 31.750±2.590 | 31.162±1.716 | >0.05 |
| Percentage of fat | 0.227±0.045 | 0.207±0.042 | >0.05 |
| Basal metabolic rate (Kcal) | 1208.000 (169.000) | 1241.000 (197.500) | >0.05 |
| Waist-hip ratio | 0.823±0.054 | 0.821±0.061 | >0.05 |
| TC (mmol/L) | 4.540 (1.295) | 4.550 (1.188) | >0.05 |
| HDL-C (mmol/L) | 1.337±0.245 | 1.452±0.338 | >0.05 |
| LDL-C (mmol/L) | 2.750 (0.800) | 2.640 (1.385) | >0.05 |
| Fasting blood glucose (mmol/L) | 5.075±0.108 | 5.084±0.636 | >0.05 |
| White blood cell count (10^9^) | 6.650 (1.775) | 7.300 (2.585) | >0.05 |
| ALT (U/L) | 18.300 (11.200) | 15.800 (12.200) | >0.05 |
| AST (U/L) | 20.900 (5.850) | 20.300 (7.450) | >0.05 |
| Serum total protein (g/L) | 75.400 (5.100) | 74.500 (11.500) | >0.05 |
| Creatinine (μmoI/L) | 58.900 (16.200) | 63.900 (19.900) | >0.05 |
| Night urine Na^+^ (mmol) | 35.608±11.285 | 29.427±12.821 | >0.05 |

Normal distribution data are shown as mean ± SD, and non-normal distribution data are expressed as median (interquartile range). Different description method has been used in this table compared with the same data in Table 1 and Table 2, they are based on the different data distribution features according to the control group. BMI, body mass index; TC, total cholesterol; HDL-C, high-density lipoprotein cholesterol; LDL-C, low-density lipoprotein cholesterol; ALT, alanine aminotransferase; AST, aspartate transaminase. n=36-37: 37 (DASH: DASH+TRE) variables between two groups.

**Supplemental Table 2. Times of aerobic and resistance exercise per week in DASH and DASH+TRE groups**

|  | **DASH** | **DASH+TRE** | ***P*** |
| --- | --- | --- | --- |
| Times of aerobic exercise (≥ 30 minutes) | 3 (3.75) | 3 (4) | >0.05 |
| Times of aerobic exercise (< 30 minutes) | 0 (1) | 0 (1) | >0.05 |
| Times of resistance exercise (≥ 30 minutes) | 0.5 (2.75) | 0 (3) | >0.05 |
| Times of resistance exercise (< 30 minutes) | 0 (0) | 0 (0) | >0.05 |

Non-normal data are shown as median (interquartile range), n=36:37 (DASH:DASH+TRE).

**Supplemental Table 3. Main nutrient composition between DASH and DASH+TRE groups after 6 weeks intervention**

|  | **DASH** | **DASH+TRE** | ***P*** |
| --- | --- | --- | --- |
| Energy (Kcal) | 2088.193±393.478 | 2109.662±321.503 | >0.05 |
| Carbohydrates (g) | 258.387± 54.050 | 256.896±43.299 | >0.05 |
| Energy from carbohydrates (Kcal) | 1033.547±216.199 | 1027.583±173.196 | >0.05 |
| Percentage of energy from carbohydrates | 0.499±0.085 | 0.488±0.0482 | >0.05 |
| Protein (g) | 101.518±26.609 | 103.262±19.992 | >0.05 |
| Energy from protein (Kcal) | 406.073±106.436 | 413.043±79.969 | >0.05 |
| Percentage of energy from protein | 0.195±0.039 | 0.196±0.020 | >0.05 |
| Fat (g) | 72.064±27.619 | 74.337±16.818 | >0.05 |
| Energy from fat (Kcal) | 648.573±248.569 | 669.037±151.364 | >0.05 |
| Percentage of energy from fat | 0.306±0.071 | 0.316±0.039 | >0.05 |

Data are shown as mean ± SD.

**Supplemental Table 4. Adherence to the dietary program**

|  | **DASH** | **DASH+TRE** | ***P*** |
| --- | --- | --- | --- |
| Days of adherence (days) | 38.194±1.721 | 38.000±2.095 | >0.05 |
| Ratio of adherence (%) | 90.938% | 90.476% | >0.05 |

Data are shown as mean ± SD.

**Supplemental Table 5. Adverse events in the DASH group**

| **NO** | **Adverse Event** | **People Counting** | **Total of occurrence** | **Proportion of occurrence** | **Severity** | **SAE**  **(Yes or No)** | **Causal relationship with the trial**  **(Yes or No)** | **Treatment measures** | **Outcome** | **Remarks** |
| --- | --- | --- | --- | --- | --- | --- | --- | --- | --- | --- |
| 1 | Nighttime hunger | 1 | 1 | 0.026 | Mild | No | No | Observe closely | Recovered | In the early stage. Similar situation before participating in this study |
| 2 | Nocturnal hypoglycemia | 0 | 0 | 0 |  |  |  |  |  |  |
| 3 | Stomachache | 0 | 0 | 0 |  |  |  |  |  |  |
| 4 | Nausea | 0 | 0 | 0 |  |  |  |  |  |  |
| 5 | Vomiting | 0 | 0 | 0 |  |  |  |  |  |  |
| 6 | Diarrhea | 0 | 0 | 0 |  |  |  |  |  |  |
| 7 | Constipation | 0 | 0 | 0 |  |  |  |  |  |  |
| 8 | Palpitations | 0 | 0 | 0 |  |  |  |  |  |  |
| 9 | Chest tightness or chest pain | 0 | 0 | 0 |  |  |  |  |  |  |
| 10 | Upper respiratory tract infection | 1 | 1 | 0.026 | Mild | No | No | Observe closely | Recovered |  |
| 11 | Other infections | 0 | 0 | 0 |  |  |  |  |  |  |
| 12 | Pain in other areas | 2 | 2 | 0.051 | Mild | No | No | Observe closely | Recovered |  |
| 13 | Fatigue | 0 | 0 | 0 |  |  |  |  |  |  |
| 14 | Withdrawal from experiment due to adverse reactions | 0 | 0 | 0 |  |  |  |  |  |  |
| 15 | Other | 0 | 0 | 0 |  |  |  |  |  |  |

SAE, Serious Adverse Events; Pain in areas other than chest pain and stomach ache.

**Supplemental Table 6. Adverse Events in DASH+TRE group**

| **NO** | **Adverse Event** | **People Counting** | **Total of occurrence** | **Proportion of occurrence** | **Severity** | **SAE**  **(Yes or No)** | **Causal relationship with the trial**  **(Yes or No)** | **Treatment measures** | **Outcome** | **Remarks** |
| --- | --- | --- | --- | --- | --- | --- | --- | --- | --- | --- |
| 1 | Nighttime hunger | 2 | 5 | 0.054 | Mild | No | Yes | Observe closely | Recovered | In the early stage; No similar situation before participating in this study |
| 2 | Nocturnal hypoglycemia | 0 | 0 | 0 |  |  |  |  |  |  |
| 3 | Stomachache | 0 | 0 | 0 |  |  |  |  |  |  |
| 4 | Nausea | 1 | 1 | 0.027 | Mild | No | No | Observe closely | Recovered |  |
| 5 | Vomiting | 0 | 0 | 0 |  |  |  |  |  |  |
| 6 | Diarrhea | 1 | 1 | 0.027 | Mild | No | No | Observe closely | Recovered |  |
| 7 | Constipation | 0 | 0 | 0 |  |  |  |  |  |  |
| 8 | Palpitations | 0 | 0 | 0 |  |  |  |  |  |  |
| 9 | Chest tightness or chest pain | 0 | 0 | 0 |  |  |  |  |  |  |
| 10 | Upper respiratory tract infection | 0 | 0 | 0 |  |  |  |  |  |  |
| 11 | Other infections | 0 | 0 | 0 |  |  |  |  |  |  |
| 12 | Pain in other areas | 0 | 0 | 0 |  |  |  |  |  |  |
| 13 | Fatigue | 0 | 0 | 0 |  |  |  |  |  |  |
| 14 | Withdrawal from experiment due to adverse reactions | 0 | 0 | 0 |  |  |  |  |  |  |
| 15 | Other | 0 | 0 | 0 |  |  |  |  |  |  |

SAE, Serious Adverse Event; Pain in areas other than chest pain and stomach ache.
