# Supplementary material for: Effects of DASH diet with or without time-restricted eating in the management of stage 1 primary hypertension: a randomized controlled trial
Source: Nutr J. 2024 Jun 17;23:65. doi: 10.1186/s12937-024-00967-9 (PMC11181626; doi:10.1186/s12937-024-00967-9)
Supplement: Supplementary file 2 — Supplementary Material 2: Supplemental Figure 1. Participant flow diagram. Participants were randomized to either DASH (n=37) or DASH+TRE (n=37).Supplemental Figure 2. Pittsburgh sleep quality index (PSQI) in the two groups. A. PSQI before and after intervention in DASH group, n=26:26, p >0.05; B. PSQI before and after intervention in DASH+TRE group, n=37:37, p >0.05. Supplemental Figure 3. Life Events Score in the two groups. A. Life Events Score before and after intervention in DASH group, n=26:26, p >0.05; B. Life Events Score before and after intervention in DASH+TRE group, n=37:37, p >0.05. Supplemental Figure 4. Body weight (A) and BMI (B) in the DASH group. n=36/time point, DASH, dietary approaches to stop hypertension. [file 12937_2024_967_MOESM2_ESM.ppt]

## Slide 1
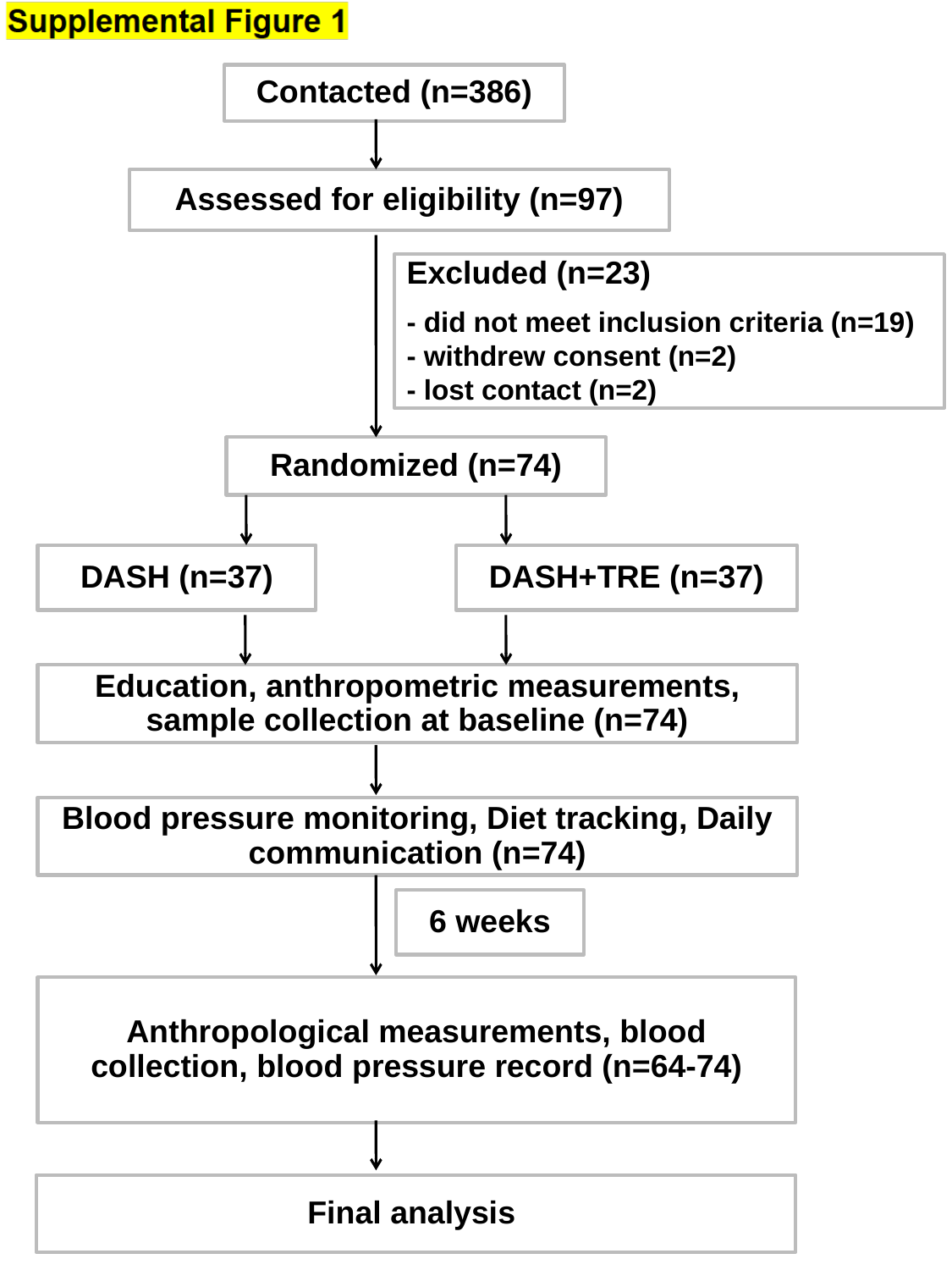

Contacted (n=386)
Assessed for eligibility (n=97)
Excluded (n=23)
- did not meet inclusion criteria (n=19)
- withdrew consent (n=2)
- lost contact (n=2)
Randomized (n=74)
DASH (n=37)
DASH+TRE (n=37)
Education, anthropometric measurements, sample collection at baseline (n=74)
Blood pressure monitoring, Diet tracking, Daily communication (n=74)
6 weeks
Anthropological measurements, blood collection, blood pressure record (n=64-74)
Final analysis

## Slide 2
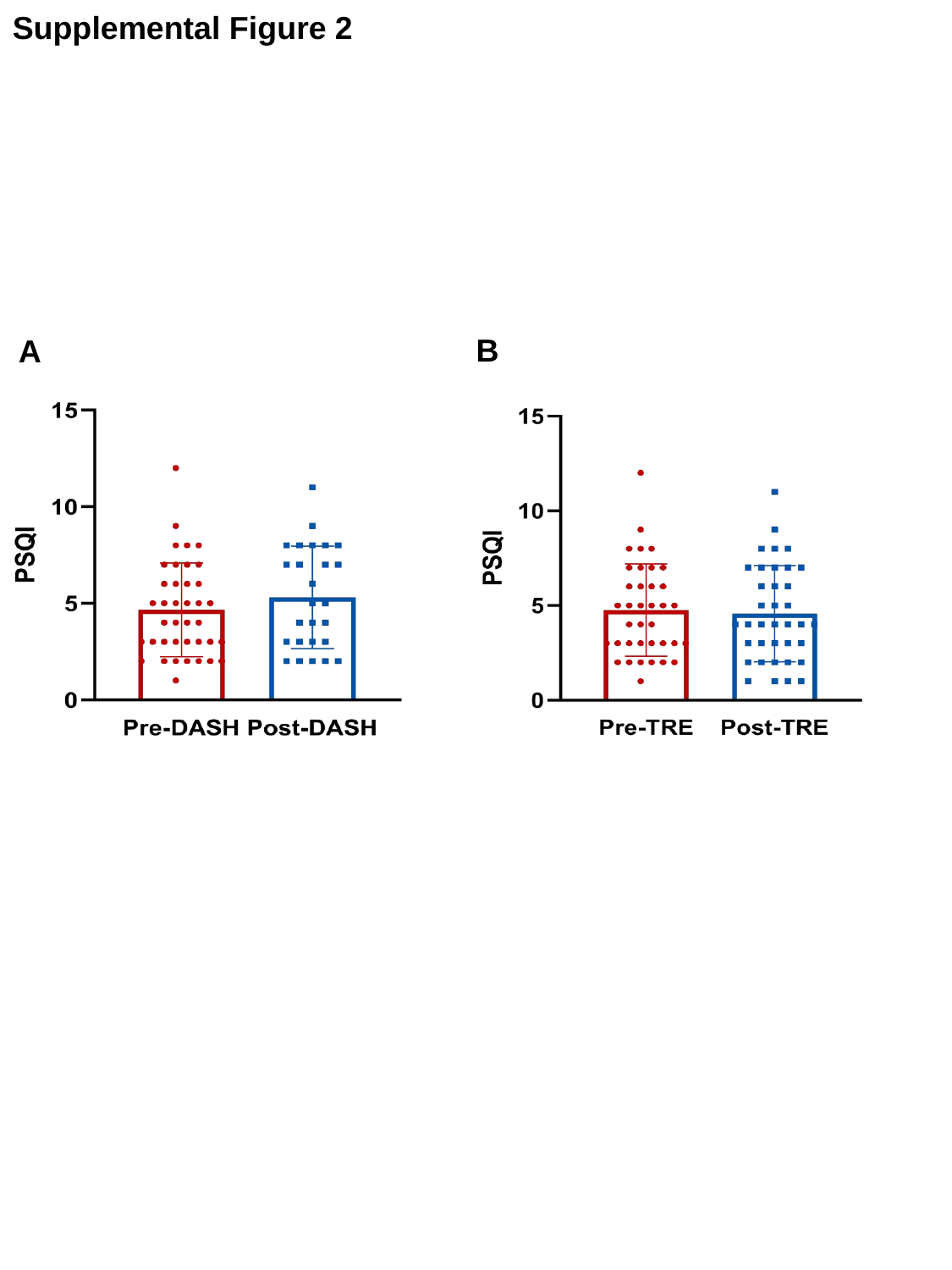

Supplemental Figure 2
B
A

## Slide 3
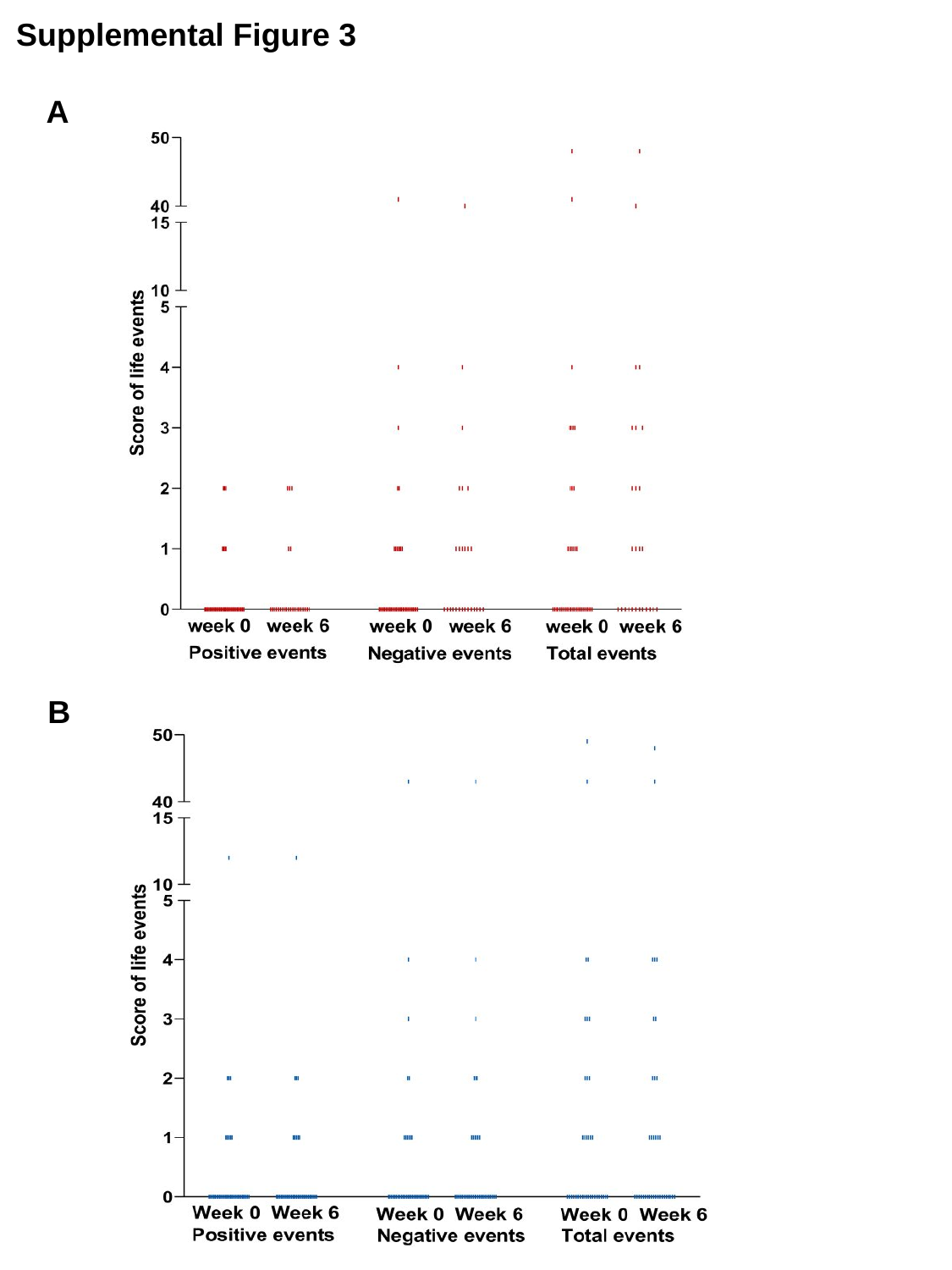

Supplemental Figure 3
A
B

## Slide 4
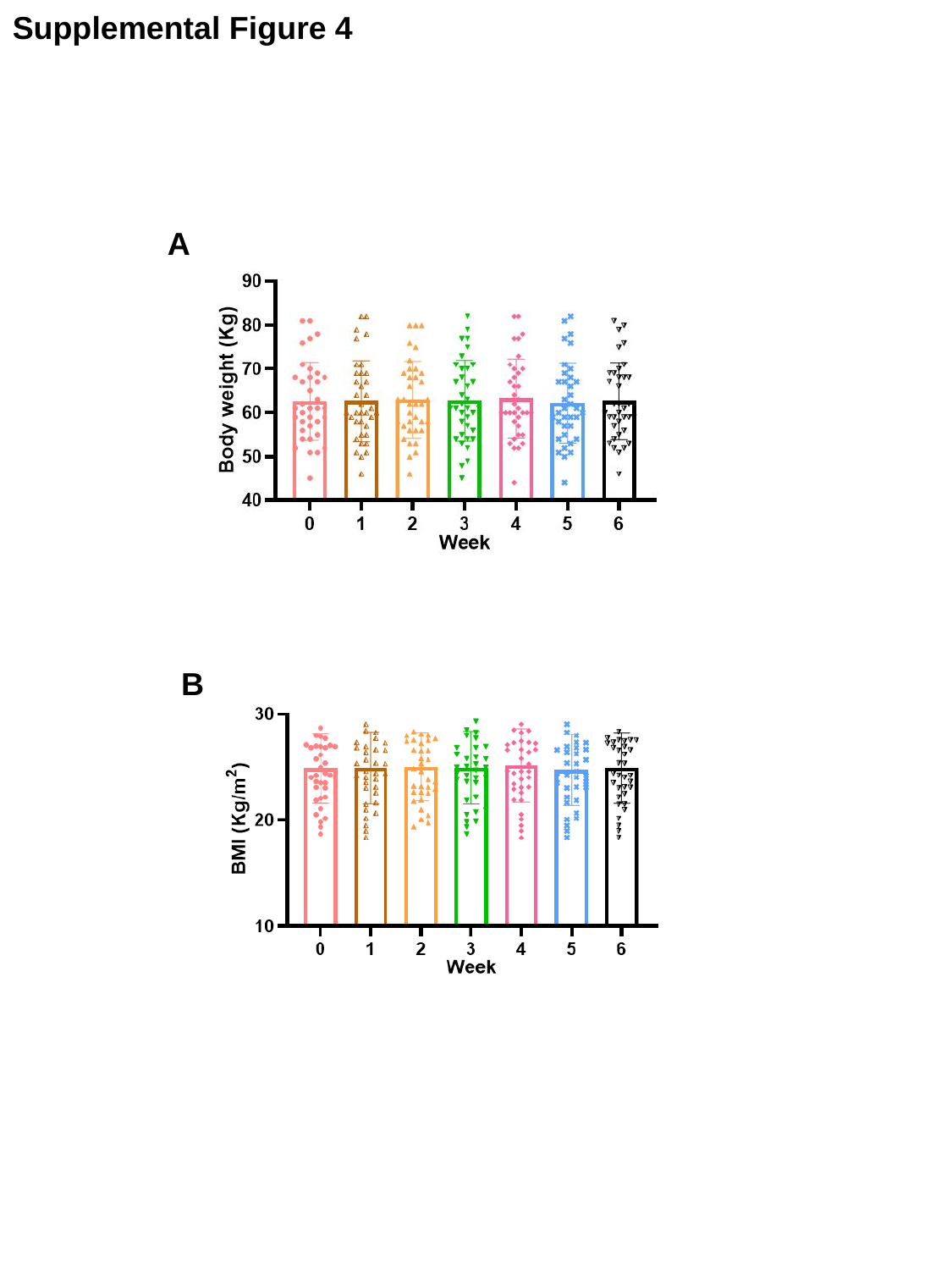

Supplemental Figure 4
A
B
